# Dynamics of Functionally Graded Laminated (FGL) Media—Theoretical Tolerance Modelling

**DOI:** 10.3390/ma16227162

**Published:** 2023-11-14

**Authors:** Jarosław Jędrysiak

**Affiliations:** Department of Structural Mechanics, Łódź University of Technology, Al. Politechniki 6, 90-924 Łódź, Poland; jarek@p.lodz.pl

**Keywords:** non-periodically laminated solids, functionally graded laminates (FGL), tolerance-periodic microstructure, microstructure effect, tolerance modelling, vibrations

## Abstract

Dynamic problems of elastic non-periodically laminated solids are considered in this paper. It is assumed that these laminates have a functionally graded structure on the macrolevel along the *x*_1_-axis and non-periodic structure on the microlevel. However, along the other two directions, i.e., *x*_2_ and *x*_3_, their properties are constant. The effects of the size of a microstructure (the microstructure effect) on the behaviour of the composites can play a significant role. This effect can be described using the tolerance modelling method. This method allows us to derive model equations with slowly varying coefficients. Some of these terms can depend on the size of the microstructure. These governing equations of the tolerance model make it possible to determine formulas describing not only fundamental lower-order vibrations related to the macrostructure of these composite solids, but also higher-order vibrations related to the microstructure. Here, the application of the tolerance modelling procedure is shown to lead to equations of the tolerance model that can be used for non-periodically laminated solids. Then, these model equations are mainly used to analyse a simple example of vibrations for functionally graded composites with non-periodically laminated microstructure (FGL). Similar problems were investigated in the framework of the homogenised (macrostructural) model (Jędrysiak et al. 2006); the resulting equations neglect the microstructure effect.

## 1. Introduction

**Figure 1:**
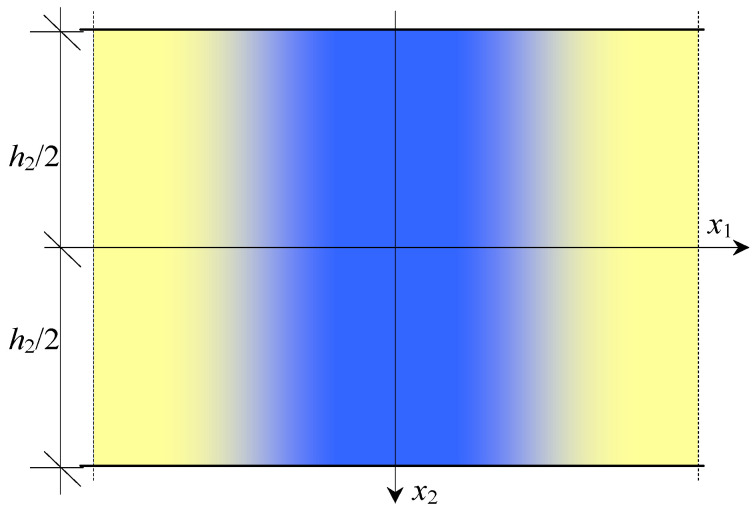
An example of a FGM-type composite.

**Figure 2:**
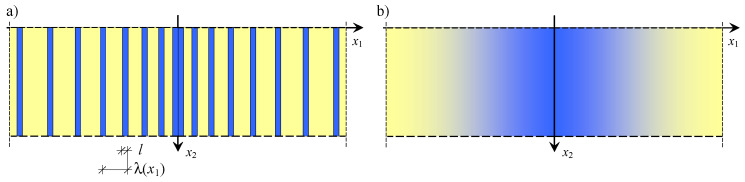
A FGL composite: (**a**) a microstructure, (**b**) a macrostructure.

Composites, having properties which change continuously, smoothly, and slowly, are called functionally graded materials (FGM) (Suresh and Mortensen [1], Woźniak et al. [2]). An example of such material is presented in Figure 1. In this paper, we considered composites on a microlevel tolerance-periodic (non-periodic) laminates along their axis *x*_1_ (Figure 2a), but, on a macrolevel, they can be treated as functionally graded along this axis (Figure 2b). Such microstructured media can be widely applied in various branches of engineering and can be named functionally graded laminated (FGL) media or, shorter, functionally graded laminates (FGL). A fragment of such a composite is shown in Figure 2.

In FGM-type composites, the distribution of properties on the macrolevel is known, but the exact description of the geometry of their microstructure is usually impossible to determine. Partial differential equations having tolerance-periodic, highly oscillating, non-continuous coefficients describe dynamics, stability, and thermal problems of FGLs under consideration. These equations do not stand as a good tool to investigate special problems. Hence, the thermomechanical phenomena in them can be considered only within the framework of mechanical models with idealised geometry. Idealising assumptions may be similar to those used in the description of macroscopic homogeneous composites. The overall behaviour of FGM materials can be analysed by adapting and modifying the methods used for homogeneous materials, although these materials are not macroscopically homogeneous. Some of the modelling methods used to describe such materials are discussed, among others, in [3] and, above all, in monographs [1,2]. Between many models, those based on the asymptotic homogenisation, the book by Bensoussan et al. [4], should be mentioned. However, these models usually neglect the microstructure effect on the general behaviour of FGLs.

Other various modelling approaches were applied in order to consider the different problems of composite media. A model with microlocal parameters used in a homogenisation was proposed for periodic plates [5].

Research on methods and models of vibrations for composite beams was presented in [6]. A modified couple stress theory with a meshless method was proposed for microstructure-dependent laminated beams in [7]. A layer-wise third order shear and normal deformable beam/plate/shell theory with geometric nonlinearity was applied to analyse finite deformations of curved laminated beams in [8]. The dynamic stability of metal foam circular plates was nonlinearly investigated in [9]. Mathematical and numerical considerations of elastic buckling for a sandwich beam, with a core of variable mechanical properties, were shown in [10]. Numerical analyses of the bending of sandwich beams with the core having inhomogeneous properties were presented in [11,12]. The dynamic stability of sandwich beams with a core of variable properties was mathematically modelled in [13]. Combined analytical-numerical models, using analytical relations and a finite element method, were formulated to consider some problems of composite auxetic beams—for a torsion [14,15], or for vibrations [16]. Carrera unified formulation with Legendre approximation was applied to analyse composite layered beams in [17].

For structures such as composite sandwich plates, various models were proposed in several papers. Stability problems for annular three-layered plates with foam cores and composite fibre-reinforced facings were considered in [18]. Free vibration analysis of sandwich plates using various modelling approaches was shown in [19]. However, a puncture problem of sandwich plates with an auxetic core was modelled in [20].

The various theoretical and numerical results of the different problems of functionally graded media were presented in a number of works. For the thermal and mechanical problems of functionally graded fibres, reinforced and laminated composites with microstructures and the higher-order theory were proposed and used in [21,22,23], and then evaluated by the finite element method in [24]. However, the known numerical methods were also applied to functionally graded materials. For the thermal analysis of composites with fibres, the boundary element method was used in [25]. The finite element method was implemented for functionally graded materials in [26].

In a series of works, the analysis of various thermomechanical problems of functionally graded beams can be found. A meshless method was used to analyse vibrations of sandwich beams that have composite functionally graded cores in [27]. The effect of the shear correction function was taken into account in the modal analysis of composite functionally graded beams in [28]. A one-dimensional theory of dynamic problems for curved laminated beams using a generalisation of layer-wise displacement approaches was formulated in [29]. An optimisation of free vibrations for composite functionally graded nano-beams was proposed in [30]. A variational approach was used to formulate a microstructure-dependent magneto-electro-elastic functionally graded porous (MEEFGP) beam models in [31]. The porosity variation of the two-phase beam model via the thickness was considered. Moreover, the extended modified couple stress theory in the proposed model was included in order to take into account the so-called microstructure effect. In [32], a new size-dependent axially functionally graded (AFG) micro-beam model was developed, in which a reformulated strain gradient elasticity theory (RSGET) was also applied. The strain gradient, velocity gradient, and couple stress effects were included in this new model for the two-component axially functionally graded beam. Hamilton’s principle was used to derive the governing equations and complete the boundary conditions of the AFG beam.

Similarly to the above, different problems of composite plates and shells, that also have a functionally graded structure, were considered in many papers. Some of them are mentioned below. Stability problems of cylindrical shells, which have functionally graded structures and were loaded by dynamic torsional loadings, were analysed in [33]. Natural frequencies for functionally graded plates were investigated using meshless methods, e.g., in [34]. Using a refined theory, a radial basis function approach for free vibrations of functionally graded plates was proposed in [35]. Free vibrations of composite shells and panels were considered when using a GDQ solution in [36]. Higher-order shear deformation theories were applied to analyse coupled thermoelasticity for FGM-type plates in [37], and statics for such plates and shells in [38]. Shells with functionally graded material properties were numerically analysed in [39], using a new low-order shell element. The effects of shear and normal deformations were taken into account for the free vibration problems of thick plates with functionally graded structure in [40]. A higher-order shear and normal deformable theory were applied to analyse the vibrations of functionally graded plates on a foundation in [41]. A refined shear deformation theory was used to consider the nonlinear analysis of bending for functionally graded plates in [42]. Chaotic dynamic problems of plates from functionally graded materials were analysed in [43]. Static problems for functionally graded shells and panels were considered in [44]. Higher-order equivalent single-layer theory was applied to analyse stress and strain recovery for functionally graded shells in [45]. The static behaviour of functionally graded shells under point and line loads was considered in [46]. A strong formulation finite element method for the statics and dynamics of laminated plates was proposed in [47]. The semi-analytical methods based on the classical laminate plate theory were formulated and used to consider some mechanical problems, e.g., for the dynamics and stability of functionally graded thin plates in [48]; for buckling of FML-FGM columns, made of such plates, with open cross-sections in [49]; similar columns with closed cross-sections in [50]; or for the imperfection sensitivity of the post-buckling of FML columns in [51]. Using the modified couple stress theory, a new plate model of the thermal buckling of functionally graded annular plates with microstructure was proposed in [52]. A strong formulation of isogeometric analysis for laminated plates was presented in [53]. The analysis of composite plates applying a differential quadrature finite element method and a layerwise theory was shown in [54]. Three-dimensional finite element modelling was used to investigate the free vibrations of FGM-type sandwich plates under thermal loads in [55]. Novel semi-analytical solutions of the transient behaviours for FGM-type plates with in-plane displacements and within a thermal environment were presented in [56]. An analytical method, based on the complex variable approach, was proposed in [57] to the analysis of moments and forces in functionally graded plates with a triangular hole.

A generalised differential quadrature method was applied to consider both the natural frequency responses of hybrid functionally graded nanocomposite shells in [58], and the effects of various reinforcements on vibrations for three-phase nanocomposite shells in [59].

Unfortunately, proposed modelling approaches for microstructured media usually lead to model equations, which neglect the microstructure effect (Brillouin [60]). Therefore, this effect cannot be investigated within these models. However, this effect may play a significant role in the overall behaviour of microheterogeneous bodies [60]. This can be seen mainly in dynamic problems, in which it is possible to observe the relationships between macro- and micro-vibrations, and the macro and microstructure of the body under consideration, respectively. Similar problems were analysed for certain media with microstructures within specially adopted methods. They can be found, for example, in [61], where the analysis of vibration band gaps in periodic beams was considered using the differential quadrature method, and in [62], where the analysis of the properties of vibration band gaps for Mindlin’s periodic plates applying a spectral element method was shown. The microstructure effect has also been experimentally confirmed for periodic laminates in [63].

Differential thermomechanical problems of microstructured media (periodic or non-periodic) can be investigated by applying an alternative approach, named *the tolerance modelling method* (or *the tolerance method*), to the monographs (Woźniak and Wierzbicki [64], Woźniak et al. (Eds.) [2,65]). This method is applicable when considering various problems of mechanics in different bodies with microstructure. The differential equations describing these problems have highly oscillating, discontinuous functional coefficients, and are not a good tool for solving such problems. The tolerance method allows for the replacing of these exact equations with averaged model equations, having constant (or slowly varying) coefficients. Some of these coefficients are directly dependent on the size of the microstructure.

Various mechanical problems of different periodic media were considered in many works using the tolerance method. Fluid-saturated micro-periodic grounds were analysed in [66]. Certain forced vibrations of periodic plates, resting on a periodically inhomogeneous Winkler foundation, were considered in [67]. Some problems of the dynamics of the plane periodic structures were investigated in [68]. Meso-shape functions applied to vibrations of wavy plates were considered in [69]. In [70], the dynamics of reinforced thin periodic plates were investigated. Some problems of the dynamics for uniperiodic medium thickness plates were presented in [71]. The length-scale effect in the buckling of periodic thin plates on a Winkler foundation was analysed in [72]. An analysis of the dynamics of periodic thin plates, with the size of the microstructure of an order of the plate thickness, was shown in [73]. Stability, dynamics, and thermoelasticity problems of micro-periodic shells were considered in papers [74,75,76,77,78,79]. Elastostatics of periodic thin plates with large deflections were investigated in [80]. The vibrations of geometrically nonlinear periodic beams were analysed in [81,82], but a comparison of natural vibration frequencies for Bernoulli and Timoshenko beams was shown in [83]. The tolerance approach was applied in order to consider vibrations for periodic sandwich plates in [84]. The tolerance modelling, in a revisiting form for dynamics and stability of periodic slender beams on a foundation, was formulated in [85]. A stress distribution in thin periodic composite plates was analysed in [86]. The heat transfer problems of periodic laminates were also investigated, e.g., for third type boundary conditions in [87]; for randomized material properties in [88].

The tolerance method is also a good tool for modelling and analysing the functionally of structures with tolerance-periodic microheterogeneity. Various aspects of the elastic responses of laminates with functional gradation of properties were shown in the works [89,90,91]. The dynamics and stability problems of longitudinally graded plates (e.g., annular plates) were considered in [92,93,94,95]. An analysis of vibrations for thin functionally graded plates with a microstructure was presented in [96], applying a new combined asymptotic-tolerance model. Vibrations of thin-walled structures with a dense system of ribs were analysed in [97,98], using a tolerance approach. Heat conduction problems in cylindrical composite media with non-uniform distributions of properties were investigated in [99,100]. The tolerance method was used in [101] to describe vibrations of functionally graded medium thickness plates with a microstructure. The dynamics and stability of functionally graded thin shells with a microstructure were considered in [102,103,104].

The above-mentioned papers do not cover the full range of problems analysed by the authors using the tolerance method for various media with a periodic or tolerance-periodic structure. They also do not constitute a complete list of such literature.

The aim of this paper is to present equations of *the tolerance model for functionally graded laminates (FGL)* and then to show an application of these equations in the analysis of a simple dynamical problem for a special example of functionally graded laminates. The example has been selected in such a way as to obtain simple analytical solutions and to show the extension of the tolerance model in relation to the asymptotic model.

This paper consists of six sections.

Section 1. ***Introduction*** includes a short description of a problem under consideration and a review of some papers related to different objects with microstructure.

In Section 2. ***Modelling preliminaries,*** basic denotations of a classical model of an elastic medium used for laminates are introduced. The known equation of such media is formulated.

Section 3. ***Modelling technique*** includes some basic concepts of the tolerance modelling method, with the reminded definitions of *the averaging operator*, *the tolerance-periodic function*, *the slowly-varying function*, *the fluctuation shape function*. In the second section, two basic assumptions of the method -*the micro-macro decomposition*, *the tolerance averaging approximation* are formulated. The third section has a short description of the tolerance modelling procedure.

In the Section 4. **Model equations,** two subsections show the governing equations of the models—the system of equations of the tolerance model of functionally graded laminates (FGL) with tolerance-periodic microstructure, and the asymptotic model of functionally graded laminates (FGL) with tolerance-periodic microstructure.

Section 5. ***An example: vibrations of a special laminated layer*** consists of three subsections. In the first section, an example under consideration of a laminated functionally graded layer on the undeformable base is described. Some basic assumptions for this example are also introduced. Vibration equations of both the models are shown in the second subsection. Equations of the tolerance model for the considered layer in the first approximation are decoupled on two equations—for the macro- and the micro-vibrations. The third section consists of three points. In the first point, it is assumed that the cell distribution functions and the properties distribution functions are defined. Equations for the macro- and the micro-vibrations are written with the boundary conditions on the upper and the bottom boundaries of the layer, and the new denotations and some additional assumptions in the second point of this subsection. Then these equations with the boundary conditions are rewritten in the non-dimensional form. In the third point, formulas of non-dimensional solutions of the macro- and the micro-vibrations are obtained. Results from these formulas are shown in the form of diagrams.

In Section 6. ***Remarks***, general and some detailed remarks of the proposed model for FGL layer and obtained numerical results of the example are formulated.

## 2. Modelling Preliminaries

For the sake of simplicity, but without losing the generality of the analysis, further considerations are limited to a plane problem of composites in the orthogonal Cartesian coordinate system *Ox*_1_*x*_2_.

Let Ω = (−*h*_1_, *h*_1_) × (0, *h*_2_) be the region of the considered laminate, where 2*h*_1_ is the dimension of the region along the *x*_1_ axis; *h*_2_ is the dimension along the *x*_2_ axis. Introduce also denotations: **x** ≡ (*x*_1_, *x*_2_); and *t* for a time coordinate. Then denote *x* ≡ *x*_1_, *z* ≡ *x*_2_. Let ∇ ≡ (∂_1_, ∂_2_), ∂ ≡ (∂_1_, 0) and $\bar{\nabla }$ ≡ (0, ∂_2_) denote derivatives of **x**, *x* and *z*, respectively. It is assumed that a distribution of laminas thicknesses is extrapolated by smooth function of cells distribution λ(*x*), being slowly varying. Let us define a closed subset ∆ of the region Ω as ∆ ≡ [−λ/2, λ/2] × {0} and the region ∆(**x**), **x**∈Ω, as ∆(**x**) ≡ **x** + ∆, such that Ω = {**x**∈Ω: ∆(**x**)⊂Ω}. The subset ∆ can be called the “basic cell” and the region ∆(**x**)—“the cell with a centre at point **x**”. Introduce also a set of centres of cells ∆(**x**) defined as Ω_∆_ = (−*h*_1_ + λ/2, *h*_1_ − λ/2) × (0, *h*_2_).

The laminate under consideration is made of two linear-elastic materials that occupy regions Ω′, Ω″, and where Γ is the system of contact planes between the components. These components are distributed in laminas with varying thicknesses. Every lamina consisted of two sub-laminas made of isotropic homogeneous materials with mass densities ρ’, ρ” and elastic tensors $C^{\prime },C^{\prime \prime }$. It is assumed that the distribution of properties is extrapolated by non-dimensional functions ν′(*x*), ν″(*x*), satisfying the condition ν′(*x*) + ν″(*x*) = 1. Moreover, the non-dimensional function $\nu =\sqrt{\nu^{\prime }\nu^{\prime \prime }}$ is introduced, which can be called a distribution function of non-homogeneity. It is also assumed that the above functions ν′, ν″ are slowly varying functions [2,89,90,91]. It can be noted that the functions adopted, defining the mass density ρ and the elasticity tensor C, are non-periodic (tolerance-periodic) functions in *x*. It is also assumed that planes *x*_α_ = const, α = 1, 2, are material symmetry planes, (i.e., *C*_1222_ = 0, *C*_2111_ = 0).

Denoting a vector of displacements by **w** = **w**(**x**,*t*) and a vector of external forces by **f**, the differential equation of the composite under consideration can be written as

(1) $$\rho \ddot{w}-\nabla \cdot (C:\nabla w)=f,$$

which is satisfied in the region Ω′∪Ω″ for every time *t*. It is assumed that the vector of displacements **w** and the stress tensor **T** = C:∇**w** satisfy regular conditions, and the condition of continuity for the stresses on contact planes between laminas Γ, i.e., E**T**F**n** = **0**, where E**T**F(**x**, *t*), **x** ∈ Γ, is a jump of the stress tensor; **n** is a unit normal vector.

The above Equation (1), describing dynamical problems of laminates with functionally graded properties (FGL), has non-periodic (tolerance-periodic), non-continuous functional coefficients. In this form, the equation is not a good tool to consider the special problems of these laminates. Hence, Equation (1) can be replaced by a system of differential equations with slowly varying, continuous functional coefficients, using the tolerance modelling method.

## 3. Modelling Technique

### 3.1. Basic Concepts

In the tolerance modelling method, some basic concepts are used, as defined in the books [2,64,65], e.g., an averaging operator <·>, slowly-varying function *SV*, tolerance-periodic function *TP*, and fluctuation shape function *FS*. They were also shown and applied in different papers, e.g., in [82], but here, some of them are reiterated, in order to make the paper self consistent [89,90,91].

Define *the averaging operator* for an integrable function *f*, determined in an interval [−*h*_1_, *h*_1_] (which can be dependent also to *z* and *t*), by

(2) $$<f>(x)=\lambda^{-1}\int_{\Delta (x)}f(y,z)dy,\;x=(x,z)\in \Omega_{\Delta },\;(y,z)\in \Delta (x).$$

The averaged value obtained from (2) for a tolerance-periodic function *f* is a slowly varying function in *x*, but this value is constant for periodic function.

Function ϕ, integrable and bounded in the region Ω, is called *the tolerance-periodic function*, ϕ∈*TP*; if for every **x**∈Ω_∆,_ there exists a function ϕ**_x_** such that the functions ϕ and ϕ**_x_** are indistinguishable within a certain tolerance, determined by the tolerance parameter δ. The function ϕ**_x_** can be named a periodic approximation of the function ϕ in the region ∆(**x**).

Function ψ, $\psi \in C(\bar{\Omega })$, is called *the slowly varying function*, ψ∈*SV*, if, and only if, the following condition is satisfied:

(∀**x**∈Ω_∆_) (∀**x**’, **x**”∈∆(**x**)) [|ψ(**x**′) − ψ(**x**″)| ≤ δ], (3)

where δ is *the tolerance parameter*, δ << 1, related to the considered problems.

Let us introduce a tolerance-periodic, bounded function φ, φ∈*TP*, defined in Ω, having a partly continuous derivative of the order 1.

Function φ is called *the fluctuation shape function*, φ∈*FSF*, depending on the parameter λ and satisfies the conditions λ∂φ∈*O*(λ) and <φ>(**x**) ≈ 0 (for every **x**∈Ω_∆_).

**Figure 3:**
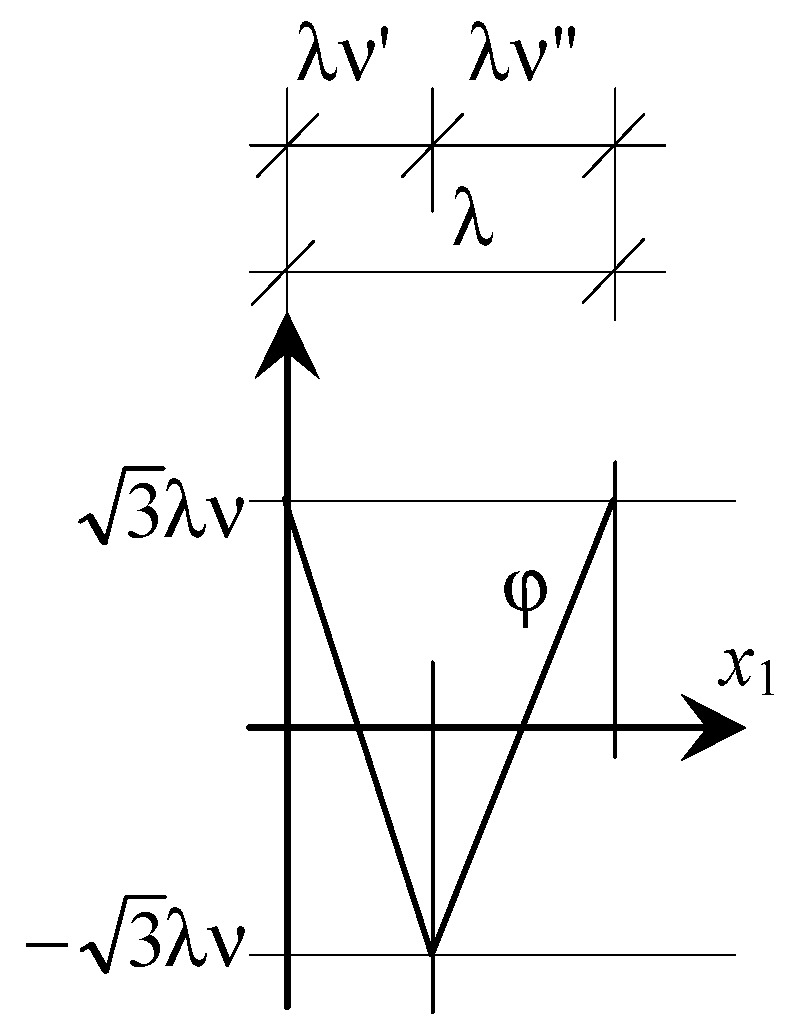
Fluctuation shape function.

In the laminates under consideration, this function is a continuous, linear function along the thickness of every sub-lamina, dependent only on the argument *x*. It is assumed in the following form:

(4) $$\varphi (x)=\left\{\begin{array}{l} -\lambda \sqrt{3}\frac{\nu (\bar{x})}{\nu^{\prime \prime }(\bar{x})}[2\frac{x}{\lambda }+\nu^{\prime }(\bar{x})]\;\mathrm{dla}\;\underset{}{x}\in (-\frac{\lambda }{2},-\frac{\lambda }{2}+\lambda \nu^{\prime \prime }(\bar{x}))\\ \lambda \sqrt{3}\frac{\nu (\bar{x})}{\nu^{\prime }(\bar{x})}[2\frac{x}{\lambda }-\nu^{\prime \prime }(\bar{x})]\;\;\;\mathrm{dla}\;x\in (\frac{\lambda }{2}-\lambda \nu^{\prime }(\bar{x}),\frac{\lambda }{2}) \end{array}\right.,\;\;\bar{x}\in (-h_{1},h_{1}),$$

and shown in Figure 3.

Because the distribution function of non-homogeneity ν is slowly varying, it can be shown that the averaged value of the function φ is equal to zero in every lamina, <φ>(**x**) ≈ 0 (it is also satisfied the condition <ρφ>(**x**) ≈ 0 for every **x**∈Ω_Δ_, where ρ > 0 is a certain tolerance-periodic function).

### 3.2. Modelling Assumptions

Using the above concepts, basic assumptions on the tolerance modelling method can be introduced [2,64,65].

In the first assumption, called *the micro-macro decomposition*, it is assumed that the displacement **w** of the composite under consideration in the form:

**w**(*x*,*z*,*t*) = **u**(*x*,*z*,*t*) + φ(*x*)**v**(*x*,*z*,*t*), (5)

where functions **u** and **v** are new kinematic unknowns, named *the macro-displacement* and *the fluctuation amplitude*, respectively. These functions satisfied the conditions

**u**(∙,*z*,*t*), **v**(∙,*z*,*t*) ∈ *SV*,

that is, they are slowly varying functions.

In the second assumption, called *the tolerance averaging approximation*, it is assumed that for any slowly varying function ψ, an approximation of the form ψ + *O*(δψ) ≅ ψ can be used, according to which quantities of the order *O*(δ) are negligibly small compared to 1.

### 3.3. Modelling Procedure

The tolerance modelling procedure was proposed in different forms [2,64,65].

Here, it is applied similarly to that shown in [2]. The first step of the tolerance modelling procedure is the substitution the micro-macro decomposition (5) into Equation (1). After this, the governing Equation (1) does not hold, hence there exists a residual field **r**(·) within the macro-dynamics, which is defined in the form

(6) $$r=\rho \ddot{w}-\nabla \cdot (C:\nabla w)-f.$$

In the next step, *the residual orthogonality condition* is formulated; it is assumed that the residual field **r**(·) has to satisfy the following conditions:

(7) <r>(x,z,t)=0,   <rφ>(x,z,t)=0.

Then, in applying the conditions (7), combined together with the modelling assumptions, a system of governing equations for the macro-displacement **u**(·,*z*,*t*) and the fluctuation amplitude **v**(·,*z*,*t*) can be obtained.

## 4. Model Equations

### 4.1. Tolerance Model Equations

From the use of the residual orthogonality condition (7) with the tolerance modelling assumptions, the averaged equations of the laminates under consideration for **u**(·,*z*,*t*) and **v**(·,*z*,*t*) are derived:

(8) $$\begin{array}{l} <\rho >\ddot{u}-\partial \cdot (<C>:\nabla u)-\bar{\nabla }\cdot (<C>:\nabla u)-\\ -\partial \cdot (v\cdot <\partial \varphi \cdot C>)-\bar{\nabla }\cdot (<\partial \varphi \cdot C>\cdot v)+<f>\underset{}{=}0,\\ \underline{<\rho {\left(\varphi \right)}^{2}>}\ddot{v}+\\ +<\partial \varphi \cdot C\cdot \partial \varphi >\cdot v-\underline{<C{\left(\varphi \right)}^{2}>}:\bar{\nabla }v+\\ +\nabla u:<\partial \varphi \cdot C>-\underline{<\varphi f>}=0. \end{array}$$

Equation (8) with the decomposition (5) stands the system of equations of *the tolerance model of functionally graded laminates (FGL) with tolerance-periodic microstructure*. The above equations have coefficients, being slowly varying functions in *x*. Moreover, some of the coefficients, underlined here, depend on the parameter λ, determining the thickness of laminas. Hence, the microstructure effect is described by Equation (8) on the overall dynamic behaviour of the considered laminates. Equation (8) is the system of differential equations for the basic unknown factors: the macro-displacement **u** and the fluctuation amplitude **v**, which have to be *slowly varying functions* in *x*. It can be observed that boundary conditions should be formulated at *x* = ±*h*_1_ only for the macro-displacement **u**, but on all other edges (at *z* = const) for every unknown: the macro-displacement **u** and the fluctuation amplitude **v**.

### 4.2. Asymptotic Model Equations

Results obtained in the framework of the above model derived using the tolerance modelling method can be evaluated by results from the approximate model. Equations of this approximate model neglect the microstructure effect. These equations can be derived using the proper asymptotic modelling procedure [2]. However, they can be also obtained directly from the tolerance model Equation (8) by neglecting the underlined terms—dependent on the parameter λ. Hence, these equations can be written as:

(9) $$\begin{array}{l} <\rho >\ddot{u}-\partial \cdot (<C>:\nabla u)-\bar{\nabla }\cdot (<C>:\nabla u)-\\ -\partial \cdot (v\cdot <\partial \varphi \cdot C>)-\bar{\nabla }\cdot (<\partial \varphi \cdot C>\cdot v)+<f>\underset{}{=}0,\\ <\partial \varphi \cdot C\cdot \partial \varphi >\cdot v+\nabla u:<\partial \varphi \cdot C>=0, \end{array}$$

which represent *the asymptotic model of functionally graded laminates (FGL) with tolerance-periodic microstructure*, omitting the microstructure effect on the overall behaviour of the laminated composites. The above equations have coefficients being slowly-varying functions in *x*, similarly to Equation (8) for the tolerance model, in the contrast to Equation (1), which have highly oscillating, tolerance-periodic non-continuous, functional coefficients.

## 5. An Example: Vibrations of a Special Laminated Layer

### 5.1. Preliminaries

**Figure 4:**
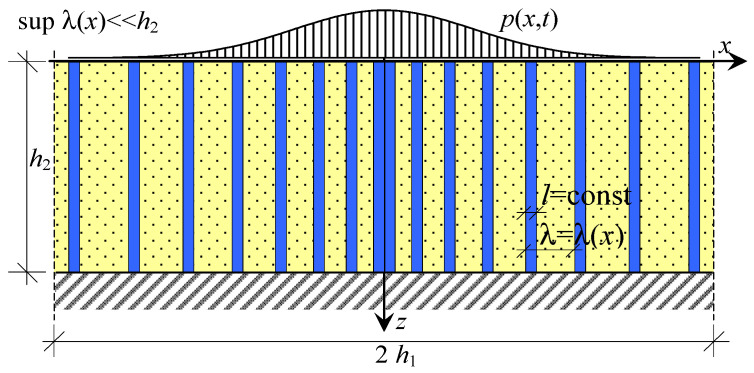
Fragment of functionally graded laminated (FGL) layer tolerance-periodically reinforced.

As an example, vibrations of a layer with the thickness *h*_2_ along the *z*-axis (*z* = *x*_2_), loaded on the upper boundary by *p*(*x*, *t*) and rested on the undeformable base are considered. The layer has the length 2*h*_1_ along the *x*-axis (*x* = *x*_1_) and is non-periodically reinforced along this axis. It is made of two components, Figure 4. It is also assumed that **f** = **0**.

Assuming for components of the macro-displacement vector *u*_1_ and the fluctuation amplitude *v*_1_, the following boundary conditions on the boundaries *x* = ±*h*_1_:

*u*_1_ = *v*_1_ = 0 and ∂_1_*u*_2_ = ∂_1_*v*_2_ = 0;

hence,

*u*_1_ = *v*_1_ = 0,

i.e., components *u*_1_, *v*_1_ are equal to zero.

Let us assume other components as independent of the argument *x*, i.e.,:

$$u_{2}(x,z,t)=u(z,t),\;\;v_{2}(x,z,t)=v(z,t).$$

### 5.2. Vibrations Equations

- The tolerance model

Under assumptions and denotations from Section 5.1, and neglecting a term with a derivative of slowly-varying coefficient $\partial_{1}\varphi <C_{1212}\partial_{1}\varphi >\approx 0$ in the first approximation in the equation for macro-vibrations (the first of Equation (8)), the governing equations of the tolerance model (8) for the FGL under consideration take the forms:

- macro-vibrations equation

(10) $$<\rho >\ddot{u}-<C_{2222}>\partial_{22}u=0;$$

- micro-vibrations equation

(11) $$<\rho {\left(\varphi \right)}^{2}>\ddot{v}+<C_{1212}{\left(\partial_{1}\varphi \right)}^{2}>v-<C_{2222}{\left(\varphi \right)}^{2}>\partial_{22}v=0.$$

It can be observed that for the considered problem of vibrations for FGL layer, Equation (8) can be written as two independent equations—one (10) for the macro-displacement *u* describing only macro-vibrations, and one (11) for the fluctuation amplitude *v* describing only micro-vibrations.

- The asymptotic model

Under assumptions and denotations from Section 5.1 and similar neglecting as in the tolerance model, the governing equations of the asymptotic model (9) for the FGL under consideration can be written as

(12) $$<\rho >\ddot{u}-<C_{2222}>\partial_{22}u=0;$$

which is only for the macro-displacement *u* and has a form identical as Equation (10). Hence, the obtained Equation (12) describes only the macro-vibrations.

### 5.3. Solutions of Special Problem of Vibrations

#### 5.3.1. Introductory Assumptions and Denotations

Let us assume the following parameters:

- the cell distribution function

(13) $$\lambda (x)=l\bar{\lambda }(x),$$

where, e.g.,:

$$\bar{\lambda }(x)=\exp{\left(\frac{x}{h_{2}}\right)}^{2}+1\;\;\mathrm{or}\;\;\bar{\lambda }(x)=\exp{\left(\frac{x}{h_{2}}\right)}^{2};$$

- for which the properties distribution functions can be written in the form:

(14) $$\nu^{\prime }(x)=\frac{1}{\bar{\lambda }(x)},\;\nu^{\prime \prime }(x)=\frac{\bar{\lambda }(x)-1}{\bar{\lambda }(x)};$$

- the loading on the upper boundary:

(15) p(x,t)=q(x,t)+r(x,t),

with the averaged part of the loading:

(16) $$q(x,t)=Q_{0}\bar{q}(x)\exp(i\omega t),$$

and the oscillating part of the loading:

(17) $$r(x,t)=R_{0}R(x)\bar{r}(x)\exp(i\omega t),$$

where $\bar{q}(x),\;\bar{r}(x)$ are slowly varying functions in *x*, called the macro-loading amplitude, the micro-loading amplitude, respectively; *R*(*x*) is an oscillating function.

The basic unknowns in Equations (10) and (11) are assumed as

(18) $$\begin{array}{l} u(z,t)=U(z)\exp(i\omega t),\\ v(z,t)=\overset{}{V}(z)\exp(i\omega t), \end{array}$$

where *U*(*z*) is the amplitude of the macro-displacement, called the macro-deflection, and *V*(*z*) is the amplitude of the fluctuation amplitude, called the fluctuation variable.

#### 5.3.2. Equations of Vibrations

Let us introduce some denotations:

(19) $$\begin{array}{l} \bar{C}\equiv <C_{2222}>=C_{2222}^{\prime }\nu^{\prime }+C_{2222}^{\prime \prime }\nu^{\prime \prime }=[C_{2222}^{\prime }-C_{2222}^{\prime \prime }+C_{2222}^{\prime \prime }\bar{\lambda }(x)]/\bar{\lambda }(x),\\ \overset{⌣}{C}\equiv <C_{1212}{\left(\partial_{1}\varphi \right)}^{2}>=12(C_{1212}^{\prime }\nu^{\prime \prime }+C_{1212}^{\prime \prime }\nu^{\prime })=12[C_{1212}^{\prime \prime }-C_{1212}^{\prime }+C_{1212}^{\prime }\bar{\lambda }(x)]/\bar{\lambda }(x),\\ \bar{\rho }\equiv <\rho >=\rho^{\prime }\nu^{\prime }+\rho^{\prime \prime }\nu^{\prime \prime }=[\rho^{\prime }-\rho^{\prime \prime }+\rho^{\prime \prime }\bar{\lambda }(x)]/\bar{\lambda }(x),\\ \beta \equiv \frac{C_{2222}^{\prime \prime }}{C_{2222}^{\prime }},\;\;\kappa \equiv \frac{\rho^{\prime \prime }}{\rho^{\prime }}, \end{array}$$

and some assumptions:

(20) $$\delta^{2}\equiv C_{2222}^{\prime \prime }/\rho^{\prime \prime }=C_{2222}^{\prime }/\rho^{\prime }=const.$$

Then let us assume the loadings amplitudes on the upper boundary as

(21) $$\bar{q}(x)=\bar{r}(x)=\frac{1-\beta +\beta \bar{\lambda }(x)}{\bar{\lambda }(x)},$$

and also β = κ. Now, introducing the following parameters:

(22) $${\bar{\omega }}^{2}\equiv \frac{\bar{C}}{\bar{\rho }h_{2}{}^{2}}=\frac{\delta^{2}}{h_{2}{}^{2}},\;\;\vartheta^{2}\equiv \frac{R_{0}\bar{\lambda }(x)\bar{r}(x)}{C_{2222}^{\prime }-C_{2222}^{\prime \prime }+C_{2222}^{\prime \prime }\bar{\lambda }(x)},$$

the equations of vibrations (10) and (11) can be written in the form:

- the macro-vibrations equation

(23) $$\partial_{22}U+\frac{\bar{\rho }}{\bar{C}}\omega^{2}U=0,$$

with the boundary conditions:

- on the upper boundary:

(24) $$\partial_{2}U(0)=-\frac{Q_{0}\bar{q}(x)}{A<C_{2222}>(x)}=-\frac{Q_{0}\bar{q}(x)\bar{\lambda }(x)}{A[C_{2222}^{\prime }-C_{2222}^{\prime \prime }+C_{2222}^{\prime \prime }\bar{\lambda }(x)]}=-\frac{Q_{0}}{AC_{2222}^{\prime }},$$

- on the bottom boundary:

(25) $$U(h_{2})=0;$$

- the micro-vibrations equation

(26) $$\partial_{22}V+\left(\frac{\bar{\rho }}{\bar{C}}\omega^{2}-\frac{\overset{⌣}{C}}{\lambda^{2}\nu^{2}\bar{C}}\right)V=0,$$

with the boundary conditions:

- on the upper boundary:

(27) $$\partial_{2}V(0)=-\frac{<\varphi^{2}>(x)}{l<C_{2222}\varphi^{2}>(x)}R_{0}\bar{r}(x)=-\frac{R_{0}\bar{r}(x)\bar{\lambda }(x)}{l[C_{2222}^{\prime }-C_{2222}^{\prime \prime }+C_{2222}^{\prime \prime }\bar{\lambda }(x)]}=-\vartheta^{2}/l,$$

- on the bottom boundary:

(28) $$V(h_{2})=0.$$

Now, the equations of vibrations (23) and (26) with the boundary conditions can be transformed to the non-dimensional form.

Introduce non-dimensional parameters:

(29) $$\begin{array}{l} \zeta \underset{}{\equiv }z/h_{2},\;\;\partial (\cdot )\equiv d/d\zeta ,\\ \Omega^{2}\equiv \omega^{2}/{\bar{\omega }}^{2},\\ \eta \equiv l/h_{2},\;\;\chi \equiv \frac{C_{1212}^{\prime }}{C_{2222}^{\prime }}=\frac{C_{1212}^{\prime \prime }}{C_{2222}^{\prime \prime }}=const,\\ \gamma^{2}\equiv \Omega^{2}-\frac{\overset{⌣}{C}h_{2}{}^{2}}{\lambda^{2}\nu^{2}\bar{C}}=\Omega^{2}-12\chi /\eta^{2}, \end{array}$$

where ζ is the non-dimensional coordinate (argument); ∂(∙) is the derivative of ζ; and some restrictions on properties of the laminated layer under consideration:

$$C_{2222}^{\prime }>>C_{2222}^{\prime \prime }\Rightarrow 1+C_{2222}^{\prime \prime }/C_{2222}^{\prime }≅1,C_{1212}^{\prime }>>C_{1212}^{\prime \prime }\Rightarrow 1+C_{1212}^{\prime \prime }/C_{1212}^{\prime }≅1.$$

Defining the non-dimensional unknowns as

(30) $$\phi (\zeta )\equiv \frac{AC_{2222}^{\prime }}{h_{2}Q_{0}}U(\zeta h_{2}),\;\;\;\psi (\zeta )\equiv \frac{\eta }{\vartheta^{2}}V(\zeta h_{2}),$$

where ϕ(ζ) is the non-dimensional macro-deflection; ψ(ζ) is the non-dimensional fluctuation variable; 

the equations of vibrations (23) and (26) take the following non-dimensional form:

- the non-dimensional macro-vibrations equation

(31) $$\partial \partial \phi +\Omega^{2}\phi =0,$$

with the boundary conditions:

(32) ∂ϕ(0)=−1,ϕ(1)=0;

- the non-dimensional micro-vibrations equation

(33) $$\partial \partial \psi +\gamma^{2}\psi =0,$$

with the boundary conditions:

(34) ∂ψ(0)=−1,  ψ(1)=0.

#### 5.3.3. Non-Dimensional Solutions of Vibrations

For the macro-vibrations described by Equation (31) the following solutions are obtained:

- the linearly decaying solution for Ω^2^ = 0:

(35) ϕ(ζ)=1−ζ;

- the oscillating solution for Ω^2^ > 0:

(36) $$\phi (\zeta )=\Omega^{-1}\left[\tan\Omega \cos\left(\Omega \zeta \right)-\sin\left(\Omega \zeta \right)\right];$$

- the resonance for:

(37) $$\Omega^{2}={\left(\frac{\pi }{2}+n\pi \right)}^{2}.$$

For the micro-vibrations described by Equation (33), the following solutions are obtained:

- the decaying solution for γ^2^ < 0, μ^2^ = −γ^2^ = 12χ/η^2^ − Ω^2^:

(38) $$\psi (\zeta )=\frac{1}{\mu }\left[\frac{\exp(-\mu \zeta )}{1+\exp(-2\mu )}-\frac{\exp(\mu \zeta )}{1+\exp(2\mu )}\right];$$

- the linearly decaying solution for γ^2^ = 0:

(39) ψ(ζ)=1−ζ;

- the oscillating solution for γ^2^ > 0:

(40) $$\psi (\zeta )=\gamma^{-1}\left[\tan\gamma \cos\left(\gamma \zeta \right)-\sin\left(\gamma \zeta \right)\right];$$

- the resonance for:

(41) $$\gamma^{2}={\left(\frac{\pi }{2}+n\pi \right)}^{2}.$$

**Figure 5:**
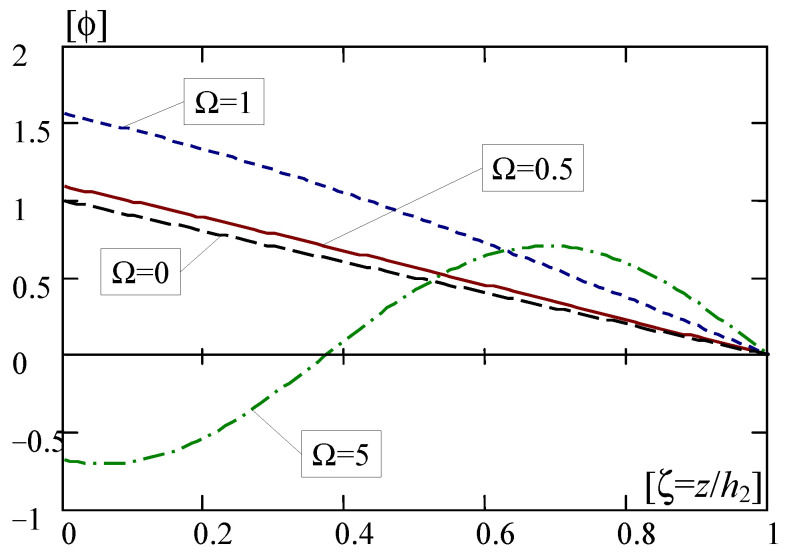
Diagrams of the non-dimensional macro-deflection ϕ (for macro-vibrations).

**Figure 6:**
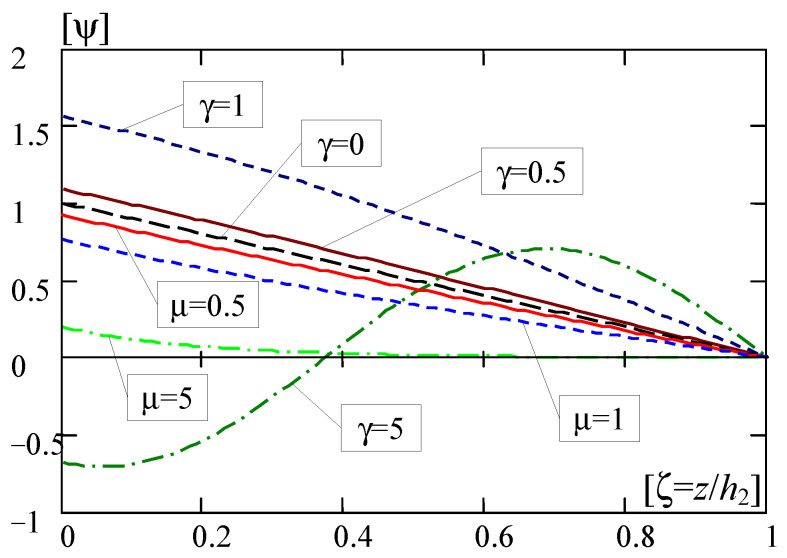
Diagrams of the non-dimensional fluctuation variable ψ (for micro-vibrations).

Some results calculated from Formulas (35), (36) and (38)–(40) are presented in Figure 5 and Figure 6, respectively, in the form of curves of the non-dimensional solutions versus the non-dimensional coordinate ζ = *z*/*h*_2_.

Figure 5 shows diagrams of the non-dimensional macro-deflection calculated using Formulas (35) and (36) for various values of parameter Ω = 0; 0.5; 1; 5.

From the solutions (35) and (36) and the above figure it can be observed that for the problem of vibrations for the FGL layer under consideration there is one linearly decaying solution of the non-dimensional macro-deflection (for Ω = 0); and are many solutions, which are decaying and oscillating (for $\Omega^{2}>0,\;\Omega^{2}\neq {\left(\frac{\pi }{2}+n\pi \right)}^{2}$). Moreover, for higher absolute values of Ω, e.g., |Ω| = 5, these solutions become strongly oscillating. The results shown in Figure 5 can be interpreted so that the macro-deflection of the layer has values other than zero at its upper free edge, subjected to load. However, on the lower edge, at the contact with the non-deformable base, the macro-deflection is zero.

In Figure 6, diagrams of the non-dimensional fluctuation variable calculated using Formulas (38)–(40) for various values of parameter γ = 0; 0.5; 1; 5 or μ = 0.5; 5 are shown.

Results calculated from the solutions (38)–(40), and presented in Figure 6, make it possible to observe that, for the considered problem of vibrations for the FGL layer, there is one linearly decaying solution of the non-dimensional fluctuation variable (for γ = 0), and are many solutions, which are decaying (for γ^2^ < 0, i.e., μ^2^ < 0), and are also many solutions, which are decaying and oscillating (for γ^2^ > 0). Moreover, for higher values of μ, e.g., μ = 5, the solutions become strongly decaying, and for higher values of γ, e.g., γ = 5, the solutions become strongly oscillating. The results shown in Figure 6 can be interpreted similarly to the macro-deflection of the layer, which means that the layer fluctuation variable has values other than zero at the upper edge of the layer, free and subject to load. However, on the lower edge, at the contact with the non-deformable base, the fluctuation variable is zero.

## 6. Remarks

In this paper, vibrations of functionally graded laminated (FGL) composites with tolerance-periodic (non-periodic) microstructures have been considered. *The tolerance modelling method* applied to the known differential equation of composites replaced this equation with tolerance-periodic (non-periodic), non-continuous coefficients by the system of governing differential equations with continuous, smooth, slowly-varying coefficients.

The microstructure effect is taken into account in the obtained averaged tolerance model equations on the overall dynamic behaviour of the composites under consideration. Thus, in these problems, this effect can be studied both at the macro and microlevels within the tolerance model.

*The asymptotic model* has been also introduced to evaluate the obtained results within the tolerance model. The governing equations of this approximate model could be derived using the asymptotic modelling procedure. This model, omitting the microstructure effect, allows the investigation of vibration problems only at the macrolevel.

In the example, it has been determined that the property distribution function in the FGL layer loaded on the upper boundary, under the condition, and the basic unknowns (the macro-displacement *u*_2_ = *u* and the fluctuation amplitude *v*_2_ = *v*), are independent of *x*_1_ = *x* coordinate.

From the example, some specific remarks can be formulated.

1. It has been shown that only the tolerance model makes it possible to investigate the effect of the micro-oscillations of the boundary loading, and also the effect of macro-oscillations.
2. The asymptotic model allows for the analysis of only the effects of the macro-oscillations of the loading.
3. Introduced various additional assumptions and restrictions make it possible to obtain the analytical formulas of solutions.
4. It can be observed that the solutions of macro-vibrations can decay linearly, decay and oscillate, or they not exist.
5. Solutions of micro-vibrations can decay exponentially, decay linearly, decay and oscillate, or they not exist.

Other more complicated and interesting problems will be investigated using the tolerance model equations of the considered FGL composites in forthcoming papers.

## Data Availability

Data are contained within the article.
